# Risk of Urticaria in Children with Type 1 Diabetes Mellitus: A Nationwide Cohort Study

**DOI:** 10.3390/ijerph17010176

**Published:** 2019-12-25

**Authors:** Shih-Yi Lin, Cheng-Li Lin, Cheng-Chieh Lin, Wu-Huei Hsu, Chung-Y. Hsu, Chia-Hung Kao

**Affiliations:** 1 Graduate Institute of Biomedical Sciences, School of Medicine, College of Medicine, China Medical University, Taichung 40402, Taiwancclin@mail.cmuh.org.tw (C.-C.L.); hsucy63141@gmail.com (C.-Y.H.); 2 Division of Nephrology and Kidney Institute, China Medical University Hospital, Taichung 40402, Taiwan; 3 Management Office for Health Data, China Medical University Hospital, Taichung 40402, Taiwan; 4 College of Medicine, China Medical University, Taichung 40402, Taiwan; 5 Department of Family Medicine, China Medical University Hospital, Taichung 40402, Taiwan; 6 Department of Chest Medicine, China Medical University Hospital, Taichung 40402, Taiwan; 7 Department of Nuclear Medicine and PET Center, China Medical University Hospital, Taichung 40402, Taiwan; 8 Department of Bioinformatics and Medical Engineering, Asia University, Taichung 40402, Taiwan; 9 Center of Augmented Intelligence in Healthcare, China Medical University Hospital, Taichung 40402, Taiwan

**Keywords:** type 1 diabetes mellitus, urticaria, National Health Insurance Research Database

## Abstract

Type 1 diabetes mellitus (T1DM) has been linked to many autoimmune problems. The association between T1DM and urticaria warrants investigation. Data were extracted from the National Health Insurance Research Database (NHIRD) of Taiwan. Participants with T1DM were recruited as the case group, and that group was matched by sex and age at a ratio of 1:4 to the control group comprising those without T1DM. The study period was 1998–2011. All participants were followed up to the diagnosis of urticaria, withdrawal from the insurance program, death, or the end of the study. A multivariable Cox proportional hazard model was used to calculate the adjusted and crude hazard ratios for urticaria. A total of 5895 participants (1179 in the case group and 4716 in the control group) were followed up in the study. The total incidence rate of urticaria in patients with type 1 DM was 26.6 per 1000 person-years, and that in controls was 6.85 per 1000 person-years. Compared with the control group, the hazard ratio of urticaria in the case group was 2.84 (95% CI = 2.27–3.56). Compared with age-matched participants without T1DM, patients with type 1 DM aged <18 years had a 3.62-fold higher risk of urticaria (95% CI = 2.85–4.59). The hazard ratio in patients with an adjusted Diabetes Complications Severity Index (aDCSI) score of 1.01–2.00 per year was 2.57 (95% CI = 1.18–5.57), and that in patients with an aDCSI score of >2.00 per year was 4.47 (95% CI = 2.68–7.47). T1DM patients aged <18 years had an increased risk of urticaria, but a similar phenomenon was not observed among T1DM patients older than 18 years.

## 1. Introduction

Urticaria, either acute or chronic, is a troublesome skin disorder with the presentation of hives and an itching sensation [1]. Acute urticaria is defined when the symptom duration is less than six weeks [2]. Acute urticaria often spontaneously resolves and has an identifiable etiology, therefore, further laboratory investigations, as conducted for chronic diseases, are not always required [3]. If the skin condition is intermittent or persistent and lasts for more than 6 weeks, it is considered chronic [4]. The manifestations of chronic urticaria are the release of histamine and proinflammatory cytokines from activated and degranulated mast cells [5]. Chronic urticaria comprises both chronic spontaneous urticaria (CSU) and chronic inducible urticaria, which includes physical and nonphysical urticaria [6]. CSU accounts for 80% of chronic urticaria cases, and autoimmunity is hypothesized to be one of the most frequent causes of CSU [7]. Confino-Cohen et al. reported that chronic urticaria is associated with autoimmune thyroid disorders, the presence of antithyroid antibodies, rheumatoid arthritis, Sjögren syndrome, celiac disease, type 1 diabetes mellitus (T1DM), systemic lupus erythematosus, and others [8]. Therefore, for chronic urticaria, laboratory workup is often required for the evaluation of its accompanying manifestation in other organs and to survey for autoimmune diseases [9].

Previous studies have indicated that chronic urticaria is less prevalent in children than in adults, and most urticaria studies have focused on adults [10,11]. However, Lee et al. showed that approximately 1.8% of children have chronic urticaria, which is comparable to the prevalence in the adult population [12]. The German birth cohort study reported that the cumulative prevalence of urticaria in children aged 10 years was 14.5% for boys and 16.2% for girls [13]. Furthermore, Fricker et al. found a higher prevalence of chronic urticarial in Asian populations vs. European and North American populations [14]. Therefore, although the prevalence of childhood urticaria has been reported [13], few studies have investigated the etiology of chronic urticaria in children.

Whether urticaria in children is also associated with autoimmune disease, similar to the phenomenon observed in adults remains unknown. Type 1 diabetes mellitus is a well-recognized autoimmune disease associated with autoantibodies in pancreatic islet cells. Therefore, we determined a type 1 diabetes mellitus disease model. We conducted a nationwide cohort study aiming to investigate the incidence of urticaria in type 1 diabetes mellitus children and adults.

## 2. Methods

### 2.1. Data Source

The National Health Insurance Administration of Taiwan established a national health insurance program in 1995. The National Health Insurance Research Database (NHIRD) of Taiwan was subsequently constructed to improve medical research resources in Taiwan. Specifically, 99% of the residents of Taiwan are enrolled into the NHI program, and their medical information is recorded in the NHIRD. Catastrophic illness data in the NHIRD were utilized to conduct the present study. The identification data for all patients were encrypted to ensure their privacy. The disease codes were recorded by physician specialists according to the International Classification of Diseases, Ninth Revision, Clinical Modification (ICD-9-CM). This study was approved by the Research Ethics Committee of China Medical University and Hospital in Taiwan (CMUH104-REC2-115-CR4).

### 2.2. Study Sample

The study cohort consisted of case and control groups. Participants with T1DM (ICD-9-CM codes 250.x1 and 250.x3) were recruited as the case group. To ensure the correct diagnosis of urticaria, we defined patients with newly diagnosed urticaria as those with at least three claims for outpatient and/or hospitalization visits. The control group included individuals without T1DM. This study excluded patients with type 2 DM (ICD-9-CM code 250), gestational diabetes mellitus (GDM) (ICD-9-CM code 648.83), and atopic diseases (allergic conjunctivitis: ICD-9-CM codes 372.05, 372.10, and 372.14; asthma: ICD-9-CM code 493; atopic dermatitis: ICD-9-CM code 691.8; allergic rhinitis: ICD-9-CM codes 477.0, 477.1, 477.2, 477.8, and 477.9; urticaria: ICD-9-CM code 708). The age criterion was younger than 30 years of age. The study period was 1998–2011. One T1DM patient was matched by sex and age to four people in the control group. The study cohort was followed up to the point of diagnosis of urticaria, withdrawal from the insurance program, death, or the end of the study.

### 2.3. Main Outcome and Covariates

The primary outcome of this study was the diagnosis of urticaria (ICD-9-CM code 708). The cohort was divided into two groups by using 18 years of age as the cutoff. The aim of this study was to investigate whether T1DM “children” had a lower or higher incidence of urticaria. Therefore, we compared T1DM patients less than and older than 18 years. However, it has been reported that chronic urticaria is associated with metabolic syndrome [15], which is prevalent among adults, especially those older than 30 years of age. To minimize the baseline bias as a result of other age-related confounding factors, we compared T1DM children and T1DM adults less than 30 years old. The urbanization level was categorized into four levels (level 1: very high population density; level 4: low population density). The NHRI stratifies all city districts and townships in Taiwan into seven urbanization levels, based on population density (people/km^2^), proportion of residents with higher education, elderly and agricultural population, and the number of physicians per 100,000 people in each area. Level 1 represents areas with a higher population density and socioeconomic status, and level 7 represents the lowest. Because few people lived in more rural areas of levels 4–7, our study included these areas in the level 4 group. [16] The categories of parental occupation were white collar, blue collar, and others, including primarily retired, unemployed, or low-income populations. A related comorbidity, inflammatory bowel disease [17,18] was also considered as a potential confounding factor.

### 2.4. Statistical Analysis

The chi-square test was performed to compare the difference in categorical variables between the case and control groups. Continuous variables were compared between the two groups using the Student’s *t* test. Incidence densities of urticaria according to each variable were calculated for the two groups. The crude hazard ratio was estimated using a univariable Cox proportional hazard model. Sex, age, urbanization level, parental occupation, and inflammatory bowel disease were included in a multivariable Cox proportional hazard model for calculating the adjusted crude hazard ratio. The effect of the adjusted Diabetes Complications Severity Index (aDCSI) score change on urticaria in T1DM patients was also analyzed. The Kaplan-Meier method was utilized to determine the cumulative incidence of urticaria for the two groups. The level of statistical significance was set at a *p* value of <0.05.

## 3. Results

A total of 5895 participants (1179 in the case group and 4716 in the control group) were followed up in the study. Table 1 presents a comparison between the case and control groups of baseline demographic characteristics and comorbidities. Approximately 53% of all participants were female. The mean age of the control group was 18.7 (±7.47) years, and that of the case group was 11.1 (±6.52) years. Almost 90% of the patients in the case group were aged below 18 years. The follow-up duration of the control group (5.78 ± 4.02 years) was longer than that of the case group (5.17 ± 3.98 years). Individuals in both groups mainly lived in an area with a high level of urbanization. In the control group, 58.3%, 29.1%, and 12.6% of parents were involved in white-collar, blue-collar, and other occupations, respectively. Parental occupation in the case group was 53.1% white collar, 30.5% blue collar, and 16.4% other. The case group showed a slightly higher incidence of inflammatory bowel disease than the control group, which is comparable to previous studies [17,18].

**Figure 1:**
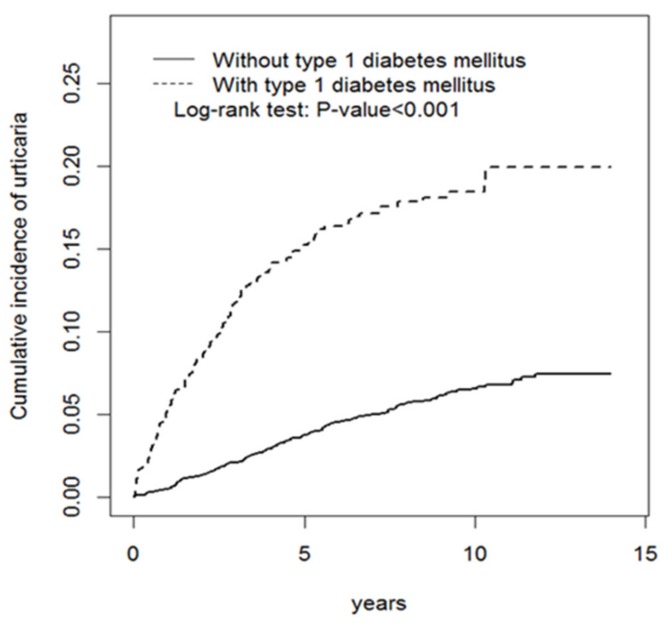
Cumulative incidence of urticaria for patients with DM (dashed line) or without type 1 DM (solid line).

As shown in Figure 1, the cumulative incidence in the T1DM cohort was significantly higher than that in the control group (*p* < 0.001). Table 2 presents the incidence densities and hazard ratio of urticaria in the case and control groups. The total incidence rate of urticaria in patients with T1DM was 26.6 per 1000 person-years, and in the control group it was 6.85 per 1000 person-years. Compared with the control group, the hazard ratio of urticaria in the case group was 2.84 (95% CI = 2.27–3.56). The risk of urticaria in female participants with T1DM was 2.05 times higher (95% CI = 1.50–2.80) than for those without T1DM, and the relative risk in male participants was 4.35 times (95% CI = 3.08–6.16). T1DM resulted in a 3.62-fold increase in the risk of urticaria in people younger than 18 years (95% CI = 2.85–4.59). In the case group, patients living in areas with urbanization levels 1–4 had a higher risk of urticaria than those in the control group; in particular, patients who lived in an area with a very high urbanization level showed the highest relative risk (adjusted HR = 4.68, 95% CI = 3.01–7.27). Regardless of parental occupation, the risk of urticaria in patients with T1DM was higher than that in controls.

The association between aDCSI score and urticaria in the case group is shown in Table 3. We considered patients with aDCSI scores of 0–0.5 per year as the reference group. The risk of urticaria significantly increased in patients with aDCSI scores over 1.01. The hazard ratio in patients with aDCSI scores of 1.01–2.00 per year was 2.57 (95% CI = 1.18–5.57), and in patients with aDCSI scores of >2.00 per year it was 4.47 (95% CI = 2.68–7.47). Patients with higher aDCSI scores had a significantly higher risk of urticaria (*p* for trend < 0.001).

## 4. Discussion

Urticaria has been reported to have a connection with autoimmune disease, such as Grave’s disease, type 1 diabetes, and autoimmune thyroiditis [19]. However, until now, there have not been cohort studies to investigate the association between autoimmune disease like type 1 diabetes mellitus and urticaria. Our results revealed an increased risk of urticaria in children with T1DM compared with children without T1DM. There are several explanations for the results. First, it has been reported that the autoimmune disease of T1DM is associated with aberrant immune responses to pancreatic β-cells, and children with T1DM might also develop other organ-specific autoimmunity [20]. Second, fluctuation of glucose levels would also activate the mast cells of the skin and release of histamine. Third, children with T1DM might have an increased likelihood of medical visits and of urticaria diagnosis compared with non-T1DM matched children. Mazzetti et al. reported T1DM diagnosis following acute urticaria, one year later with positive anti-glutamic acid decarboxylase antibodies [21]. Hyman et al. also reported diagnosis of T1DM and autoimmune thyroid disease following presentation of urticaria in children [22]. Furthermore, Brunetti et al. demonstrated that up to 31% of children with chronic urticaria had functionally active autoantibodies directed against the IgE or IgE receptor [23]. Therefore, we hypothesized that increased autoimmunity in children with T1DM might increase the likelihood of urticaria development. The increased incidence of urticaria in children with T1DM might be caused by insulin injections. Shore et al. described a patient who developed chronic urticaria after injection with porcine isophane insulin [24]. Sackey et al. reported that a 6-year-old boy with T1DM had recurrent urticaria at insulin injection sites [25]. Furthermore, the reduction in blood glucose levels might also be a trigger of urticaria. Kushe et al. reported that urticaria might be precipitated by glucose-lowering medication, as the marked reduction of blood glucose can cause a drop in body temperature, which serves as cold stimuli [26]. Besides, T1DM children have been reported as having more frequent hypoglycemic episodes and poor glycemic control [27]. As confirmed by a literature review, our study is the first nationwide-cohort study that demonstrates an association between T1DM and urticaria in children. Furthermore, our data demonstrates that T1DM is a significant risk factor for urticaria, regardless of urbanization level, income, and presence of inflammatory bowel disease; factors that have also been reported to be related to urticaria [28,29]

A similar association between T1DM and urticaria was not observed in adults in this study. This may be because we excluded those diagnosed with urticaria before enrollment into the study. It has been estimated that 15–23% of adults have experienced at least one episode of urticaria [12]; thus, certain individuals might have had urticaria before T1DM diagnosis. Those who had T1DM and concomitant urticaria in childhood were not included in this study. Therefore, adults with T1DM would not have a significantly higher incidence of urticarial than non-T1DM matched adults. Our finding that the cluster incidence of urticaria in T1DM children rather than T1DM adults might also reflect the close time relationship between urticaria and T1DM. This finding again strengthened the speculation that autoimmunity plays a role in pathogenesis for urticaria.

This study has several limitations. First, information on the results of autoantibodies, total and specific IgE, differentiated blood count, erythrocyte sedimentation rate, C-reactive protein, autologous serum skin test, thyroid hormones and thyroid autoantibodies, antinuclear autoantibodies, surveys for infection and intolerance of specific food, urine analysis, skin prick tests, and investigation of autoimmune diseases is not available in the NHIRD. However, in our preliminary analysis, we considered and adjusted for many autoimmune diseases, including autoimmune thyroid disease, Addison disease, vitiligo, ankylosing spondylitis, inflammatory bowel disease, pernicious anemia, multiple sclerosis, systemic lupus erythematosus, rheumatoid arthritis, Sjögren syndrome, myasthenia gravis, and others. We excluded the aforementioned autoimmune diseases in our formal study because the case numbers were too few and nonsignificant. Second, we did not obtain detailed data through patient questionnaires, diaries, and calendars. Third, we used ICD-9 codes for ensuring the diagnosis of urticaria, which made it difficult to differentiate between acute or chronic urticaria and identify urticaria subtypes. Therefore, we could not perform further analysis based on the subtype of urticaria, such as physical urticaria, cold urticaria, or food urticaria, which is most associated with type 1 diabetes. Fourth, we matched gender and index year between study and control cohort. Therefore, the mean ages of these two cohorts were significantly different. Finally, we did not adjust for medical visits in our analysis. Because T1DM patients have a higher likelihood of utilizing medical care, surveillance bias may exist.

## 5. Conclusions

Our study showed that T1DM patients aged <18 years had an increased risk of urticaria, but a similar phenomenon could not be observed among T1DM patients older than 18 years. Our results indicate the possibility of urticaria development in children with T1DM.

## Figures and Tables

**Table 1:** Comparison of demographic characteristics and comorbidities in patient with and without type 1 DM.

Variables	Controls	T1DM	*p*-Value
	(N = 4716)	(N = 1179)	
	*n*(%)	*n*(%)	
Sex			0.99
Female	2492(52.8)	623(52.8)	
Male	2224(47.2)	556(47.2)	
Age, years, mean (SD) a	18.7(7.47)	11.1(6.52)	<0.001
Stratified by age			<0.001
<18	2465(52.3)	1054(89.4)	
≥18	2251(47.7)	125(10.6)	
Follow up duration, years (SD)	5.78(4.02)	5.17(3.98)	<0.001
Urbanization †			0.02
1. (Very high)	1247(26.7)	350(29.7)	
2. (High)	1401(29.7)	374(31.7)	
3. (Moderate)	934(19.8)	204(17.3)	
4. (Low)	1124(23.8)	251(21.3)	
Parental occupation			0.001
White collar	2750(58.3)	626(53.1)	
Blue collar	1371(29.1)	360(30.5)	
Others ‡	595(12.6)	193(16.4)	
Comorbidity			
Inflammatory bowel disease	48(1.02)	20(1.70)	0.05

Chi-Square Test, ^a^ Student t-test. ^†.^ The urbanization level was categorized by the population density of the residential area into 4 levels, with level 1 as the most urbanized and level 4 as the least urbanized.^‡.^ Other occupations included primarily retired, unemployed, or low-income populations.

**Table 2:** Comparison of incidence densities of urticaria and hazard ratio between those with and without type 1 DM by demographic characteristics and comorbidity.

Variables	Controls			T1DM				Adjusted HR ^†^ (95% CI)
	Event	PY	Rate ^#^	Event	PY	Rate ^#^	Crude HR(95% CI)	
All	187	27,281	6.85	162	6092	26.6	3.81(3.09, 4.70) ***	2.84(2.27, 3.56) ***
Sex								
Female	127	13,863	9.16	73	3190	22.9	2.48(1.86, 3.31) ***	2.05(1.50, 2.80) ***
Male	60	13,419	4.47	89	2903	30.7	6.64(4.78, 9.21) ***	4.35(3.08, 6.16) ***
Stratified by age								
<18	121	15,992	7.57	158	5466	28.9	3.63(2.87, 4.60) ***	3.62(2.85, 4.59) ***
≧18	66	11,290	5.85	4	626	6.39	1.10(0.40, 3.02)	1.10(0.40, 3.03)
Urbanization								
1. (Very high)	41	7463	5.49	59	1861	31.7	5.74(3.85, 8.55) ***	4.68(3.01, 7.27) ***
2. (High)	58	8220	7.06	52	2004	26.0	3.61(2.48, 5.25) ***	2.51(1.68, 3.76) ***
3. (Moderate)	44	5309	8.29	25	1031	24.3	2.90(1.78, 4.74) ***	2.14(1.28, 3.56) **
4. (Low)	44	6290	7.00	26	1197	21.7	2.99(1.84, 2.87) ***	2.32(1.39, 3.87) **
Parental occupation								
White collar	110	16,133	6.82	94	3401	27.6	3.99(3.03, 5.25) ***	2.93(2.18, 3.95) ***
Blue collar	54	7513	7.19	47	1993	23.6	3.28(2.22, 4.85) ***	2.63(1.74, 3.97) ***
Others ‡	23	3435	6.33	21	697	30.1	4.25(2.34, 7.71) ***	2.60(1.35, 5.01) **
Inflammatory bowel disease								
No	185	26,820	6.90	160	5957	26.9	3.82(3.09, 4.72) ***	2.83(2.26, 3.56) ***
Yes	2	462	4.33	2	136	14.8	3.62(0.51, 25.8)	3.94(0.38, 40.5)

Rate ^#^, incidence rate, per 1000 person-years; Crude HR, relative hazard ratio; Adjusted HR ^†^: multivariable analysis including sex, age, urbanization, parental occupation, and inflammatory bowel disease. ** *p* < 0.01, *** *p* < 0.001. Others ^‡^: occupations included primarily retired, unemployed, or low-income populations.

**Table 3:** Incidence and HRs of aDCSI change for urticaria in T1DM Cohort.

Change in aDCSI Score per Year	N	No. of Events	Rate ^#^	Crude HR	95% CI	Adjusted HR ^†^	95% CI
T1DM							
0–0.50	1075	130	22.7	1	(Reference)	1	(Reference)
0.51–1.00	42	7	38.3	1.39	(0.65, 2.97	1.26	(0.59, 2.71)
1.01–2.00	28	7	96.1	3.14	(1.46, 6.75) **	2.57	(1.18, 5.57) *
>2.00	34	8	174.1	6.86	(4.18, 11.2) ***	4.47	(2.68, 7.47) ***
*p* for trend				<0.001		<0.001	

Rate ^#^, incidence rate, per 1000 person-years; Crude HR relative hazard ratio; Adjusted HR ^†^: multivariable analysis including sex, age, urbanization, parental occupation, and inflammatory bowel disease. * *p* < 0.05, ** *p* < 0.01, *** *p* < 0.001.
